# A Cross-Sectional Study to Assess Knowledge of COVID-19 among Undergraduate Students in North-Central Ecuador

**DOI:** 10.3390/ijerph18168706

**Published:** 2021-08-18

**Authors:** David Ortega-Paredes, César Marcelo Larrea-Álvarez, Santiago Isaac Jijón, Karen Loaiza, Miroslava Anna Šefcová, Gabriel Molina-Cuasapaz, Pedro Barba, Christian Vinueza-Burgos, Esteban Fernandez-Moreira, Hégira Ramírez, Marco Larrea-Álvarez

**Affiliations:** 1 Unidad de Investigación de Enfermedades Transmitidas por Alimentos y Resistencia a los Antimicrobianos (UNIETAR), Facultad de Medicina Veterinaria y Zootecnia, Universidad Central del Ecuador, Quito 170129, Ecuador; daortegap@gmail.com (D.O.-P.); cvinueza@uce.edu.ec (C.V.-B.); 2 Facultad de Ciencias Médicas Enrique Ortega Moreira, Carrera de Medicina, Universidad Espíritu Santo, Km 2.5 vía Samborondón 0901952, Ecuador; esteban.f.moreira@gmail.com; 3 Research Unit Life Science Initiative (LSI), Quito 170102, Ecuador; cmla88@hotmail.com (C.M.L.-Á.); karenloaizaconza@gmail.com (K.L.); miroslava.sefcova@gmail.com (M.A.Š.); 4 School of Biological Science and Engineering, Yachay-Tech University, Hacienda San José, Urcuquí 100650, Ecuador; santiago.jijon@yachaytech.edu.ec; 5 Facultad de Ciencias Agropecuarias y Recursos Naturales, Carrera de Medicina Veterinaria, Universidad Técnica de Cotopaxi, Latacunga 050101, Ecuador; edie.molina7278@utc.edu.ec; 6 Carrera de Biotecnología, Facultad de Ingeniería en Ciencias Agropecuarias y Ambientales, Universidad Técnica del Norte, Ibarra 100105, Ecuador; pmbarba@utn.edu.ec; 7 Escuela de Medicina, Universidad de Las Américas, Quito 170517, Ecuador

**Keywords:** COVID-19, SARS-CoV-2 virus, knowledge, awareness, undergraduate students, Ecuador

## Abstract

Adherence to preventive measures is influenced by people’s knowledge, attitudes and practices towards a disease; therefore, assessing knowledge of COVID-19 is critical in the overall effort to contain the outbreak. This cross-sectional study was conducted among undergraduates (*n* = 3621) of different programs and different levels of education associated with universities in north-central Ecuador. The form consisted of 32 questions covering demographics, symptoms, detection, treatment, transmission, prevention and knowledge of the virus. The rate of correct answers was 75.5% (21.1 ± 5 out of 28), with differences observed regarding program of study, educational level and location of institution (α = 0.05), although effect size analyses showed that these differences could not be considered large. Multiple linear regression analyses showed that lower scores were associated with initial stages of education, careers related to social sciences and location of institution. Participants possessed sufficient knowledge about detection, transmission and prevention, although they overestimated fatality rate and were less confident about the characteristics of the virus and the effectiveness of traditional medicine. Consequently, future educational programs must place emphasis on addressing deficient knowledge. Certainly, improving COVID-19 literacy will promote the appropriate application of protective measures aimed at preventing the virus’ spread.

## 1. Introduction

The coronavirus disease 2019 (COVID-19) was initially detected in late December 2019 in China and has since spread worldwide. By May 2021, more than one hundred million cases had been confirmed, and at least 3.7 million deaths had been attributed to COVID-19 [1]. The virus responsible for COVID-19, SARS-CoV-2, belongs to the coronavirus family, members of which are considered zoonotic pathogens as they can cause and transmit diseases between various animal species, including humans, bats, cats or camels [2]. Coronaviruses are positive-sense single-stranded RNA viruses that have been classified according to their antigenicity into four groups: alpha, beta, gamma and delta [3]. All of these groups are known to infect mammals, but beta coronaviruses are capable of infecting humans [4]. SARS-CoV-2 causes mild respiratory tract infections and influenza-like symptoms, accompanied by lung injury, multi-organ failure and potentially death [5,6].

Unlike its predecessors, SARS-CoV-2 is highly contagious due to its spread mechanisms via direct contact or respiratory droplets. This has contributed to its rapid transmission and the vast number of confirmed cases [7,8]. Public health measures have not been able to decrease the dissemination of the virus. This is particularly relevant in areas where medical facilities are not adequate for coping with such a devastating disease [9], including most of the global south, especially Sub-Saharan Africa and Latin America [10,11]. In Ecuador, the first case of COVID-19 was reported in March 2020; the consequences of this disease have since affected the country, with confirmed cases in all 24 provinces [12]. Nationally, the case fatality rate, until the 8th of June 2021, was around 4.8%, with 20,831 deaths out of 432,985 cases [12].

Critical misconceptions concerning COVID-19 repeatedly circulate on social media, not only regarding transmission, treatment and prevention but also about the origin and characteristics of the virus. Some common misunderstandings that have gained popularity worldwide include the role of wild animals in transmission or the use of antibiotics and saline solutions for COVID-19 prevention [13,14,15]. Undoubtedly, these misunderstandings could have a negative impact on the public [1] as these errors may contribute to undermining public adherence to control and prevention campaigns, which are known to be influenced by people’s knowledge, attitudes and practices regarding disease [16,17]. In Ecuador, several misconceptions have been extensively disseminated on social media, especially those about the origin of the virus, development of vaccines and unproven treatments, including the use of seawater, ginger or eucalyptus [18,19]. Preventive strategies and health programs must address deficient knowledge of contagious diseases. However, assessing COVID-19 knowledge is crucial because it provides reference information useful for defining the actions required to counteract the effects of the outbreak.

Previous studies assessing knowledge of COVID-19 in Ecuadorians have reported a correct response rate of around 80% [20,21], which can be considered moderate [22,23,24]. It could be argued that COVID-19 literacy is not necessarily proficient in the area. In general, Ecuadorian citizens demonstrated knowledge about symptoms, detection, transmission and prevention of the disease [20,21]. However, participants not only agreed with misconceptions regarding the use of unproven and traditional treatments but also lacked a proper understanding of the virus’ origins and composition [20]. In line with these results, a previous study revealed that students in Quito, Ecuador, who were not following careers related to life sciences were unfamiliar with fundamental genetic concepts such as genes carrying protein-making information or the number of chromosomes passed down to the next generation [25]. It could be argued that basic genetic information is not necessarily popular among people with a college education, which seems particularly relevant in the context of COVID-19. For instance, appropriate knowledge of RNA structure and function appears fundamental for understanding the use of RNA vaccine technology. Likewise, knowledge about the mechanisms by which the virus could have originated via natural selection might help people adhere to preventive practices that reduce the likelihood of future zoonotic events [20].

Little information is available regarding COVID-19 knowledge among undergraduate students. This is dangerous because, in Ecuador, the national educational institutions have suggested reopening plans [26], and doing so could increase the number of infections. One study showed that medical students affiliated with a single university in Quito were knowledgeable about prevention, practices and symptoms [27]. However, no information is available for other regions of the country. A large number of students are enrolled in universities located in the northern Sierra region of the country, principally in the cities of Quito, Ibarra, and Latacunga. This study aimed to assess the knowledge of undergraduate students toward COVID-19 at universities located in these cities. Understanding their awareness towards COVID-19 will constitute a reference for authorities in charge of planning and executing educational programs and health campaigns.

## 2. Materials and Methods

### 2.1. Study Instrument

The questionnaire applied in this study has been previously used to assess knowledge of COVID-19 in Ecuadorian citizens tested for virus knowledge in Quito and Ibarra, with a Cronbach alpha coefficient of 0.65 [20]. Coefficients varying from 0.6 to 0.7 are considered reliable and adequate [23,28], so the formulated questions seemed acceptable for measuring COVID-19 knowledge. The questionnaire consisted of 32 questions divided into seven sections (Table S1). The first part included demographic data such as age, sex, educational level (1st–3rd, 4–6th, 7–9th, 10th or higher semester), program of study (applied sciences, social sciences) and location of the institution (Quito, Ibarra or Latacunga). Applied sciences included all programs considered to be science-related. Namely, all careers employing the scientific method to acquire and process data, such as engineering, biology, chemistry or medicine. All careers devoted to the study of societies and their individuals were grouped under the name “social sciences”, which included economics, politics, linguistics, history and others. From this point, inquiries were enumerated from 1 to 28. The second part consisted of questions concerning symptoms; the third comprised questions regarding diagnostic tests; the fourth covered knowledge about treatments; the fifth section focused on transmission; while the sixth focused on prevention strategies. The final section was devoted to evaluating knowledge about the SARS-CoV-2 virus, including its characteristics, origin and composition. In addition, people were offered a section at the end of the form where they were able to share their thoughts, impressions and recommendations. True or False (T/F) questions were provided with three answer options: “true”, “false” and “not sure”; multiple-choice questions had only one acceptable answer. Correct responses obtained a point, while incorrect or “not sure” replies did not. Summed scores ranged from 0 to 28, with higher scores denoting superior knowledge.

### 2.2. Study Design and Procedure

The on-line sample size calculator “Raosoft” was used to calculate the sample size [29], which was estimated with a response distribution of 50%, a confidence level of 99%, and a margin of error of 5%. As there are no official data on the number of undergraduate students per region, we used the information provided by each university on their webpages. Based on these data, we estimated the total undergraduate population of north-central Ecuador to be around 250,000. The required sample was 662, the true sample was 3621, which is 5.5 times larger than the estimated value. The present cross-sectional study was conducted from the 15 June to the 15 August, 2020, among undergraduate students affiliated with various universities in the Ecuadorian cities of Quito, Ibarra and Latacunga. The on-line survey was generated in Spanish on Google Forms and sent to students via the official channels of communication for each university. Participants were tutored to complete the questionnaire by following the instructions provided; the correct answers were revealed after completing the questionnaire. The selection of individuals was based on non-random criteria, as this investigation sought to develop a primary understanding of COVID-19 knowledge among undergraduates based on the northern Sierra region of the country. The present research did not aim at testing any hypothesis about a larger population, so it was carried out using voluntary responses. Additionally, to enhance the reliability of the survey, we referred to the checklist for reporting results of internet e-surveys (CHERRIES) (Table S2).

### 2.3. Participants

The questionnaire was appropriately answered by 3621 undergraduate students affiliated with various universities in north-central Ecuador. The mean age of participants was 22.7 ± 5 years, ranging from 17 to 28. The number of female students exceeded that of males, and most participants claimed to pursue a career in applied sciences. The majority of interviewees were freshmen (1st–3rd semesters, 4–6th semesters), followed by junior (7–9th semester) and senior students (10th or higher). Students were mainly associated with universities in Ibarra, followed by those affiliated with institutions in Quito and Latacunga.

### 2.4. Ethical Considerations

The ethics committee of the human research office of scientific integrity of the Universidad de las Américas (CEISH-UDLA) revised and accepted the protocol (Reference number: OIC-CEISH-UDLA-2020-03-23-013). All procedures involving human participants were conducted in accordance with the Declaration of Helsinki. Each institution that consented to collaborate in the study reviewed and approved the survey. The questionnaire was voluntary, anonymous and confidential; students could withdraw from the session at any point, if desired. The information gathered did not expose students to any dangerous situations, and no identifiable data were collected. Informed consent was obtained from all participants prior to their involvement in the study.

### 2.5. Statistical Analysis

Frequencies and percentages were used for data description. Score comparisons by sex and study program were carried out using Welch’s *t*-test, whereas an unbalanced ANOVA was utilized to compare scores by educational level. Welch’s ANOVA was used to compare scores by location of institution; these procedures were carried out along with a Tukey post-hoc. Levene’s test showed that the data regarding “sex”, “program” and “location of institution” were heteroscedastic, while data involving “level of education” were homoscedastic. Thus, different ANOVAs were used for comparing scores by “level of education” (unbalanced ANOVA) and “Location of institution” (Welch’s ANOVA). Furthermore, Welch’s *t*-test was applied for comparing data by sex and program of study. The *p*-values obtained with the aforementioned methods helped to determine differences between demographic variables. However, to measure if differences were small, medium or large, effect size measures were estimated for each test, along with their confidence intervals. Cohen’s d was determined for the *t*-test and Tukey post-hoc analyses, where values of 0.2 were interpreted as small differences, while values around 0.5 and 0.8 denote medium and large differences, respectively. Partial Eta Squared (*η*^2^) was calculated for ANOVA, where values around 0.01, 0.06 and 0.14 show small, medium and large differences, respectively [30,31,32,33]. Multiple linear regression analysis was used to detect potential factors associated with lower scores in demographic categories, independent variables and the dependent variable, as described in [24]. Factors were selected using a stepwise method, as this approach permits the addition and removal of terms from a linear model based on their significance in explaining the response variable. The final model contained only variables that proved to be significant. The association between predictors was assessed with chi-square, which revealed significant associations. However, these associations were considered low due to the values of Cramer’s V. The stepwise regression did not consider terminus of interaction. Questions answered correctly by less than 50% of the population were selected for further analysis. Chi-square was used to determine possible relations between these questions (Q8, Q23, Q24, Q26 and Q28) and demographic variables. Statistical significance was set at *p* < 0.05. Analyses were performed in MATLAB^®^ version 9.9.9341360 (MathWorks, Natick, MA, USA) (R2016a); figures were rendered with Python’s plotting library, Matplotlib 3.0.3 (Python Software Foundation, Fredericksburg, VA, USA).

## 3. Results

### 3.1. Demographics and Overall Scores

The overall mean score was 21.16 ± 3 out of 28, with a correct rate of 75.57%; significant differences were detected with regards to the study program, educational level and location of institution (*p* < 0.001). A total of 33 individuals (0.91%) obtained the maximum score. From this group, 22 were males, 11 females, 16 freshmen, 8 juniors and 9 seniors. Twenty-three were enrolled in careers related to applied sciences, while ten belonged to disciplines in social sciences. Twenty-two were affiliated to universities in Quito, ten to institutions in Ibarra and one to universities in Latacunga. Students enrolled in applied science careers scored higher than those studying social sciences; the effect size analysis showed that the assessed differences could be considered medium (Table 1). Likewise, students from higher semesters (7th onwards) had higher scores than freshmen (1st–6th semester) (Table 1). Values of Cohen’s d revealed that the estimated differences could be regarded as medium (Table 2). Students from universities in Quito scored higher than the rest (Table 1), although these differences could not be considered large (Table 2).

Multiple linear regression (R^2^ = 0.15) showed that careers in social sciences (vs. careers in applied sciences, β: −1.39, *p* = 0.015), freshman levels (1st–3rd semester; 4–6th semester) (vs. 10th semester or higher, β: −1.75; −0.79, *p* < 0.001), junior level (7–9th semester) (vs. 10th semester or higher, β: −0.48, *p* < 0.001), Ibarra (vs. Quito, β: −1.62, *p* < 0.001) and Latacunga (vs. Quito, β: −2.19, *p* < 0.001) were associated with lower scores (Table 3). The ANOVA analysis showed that male students obtained slightly higher results than their female counterparts (Table 1), although the final multiple regression model did not retain sex as a predictor.

### 3.2. Knowledge of COVID-19

**Figure 1:**
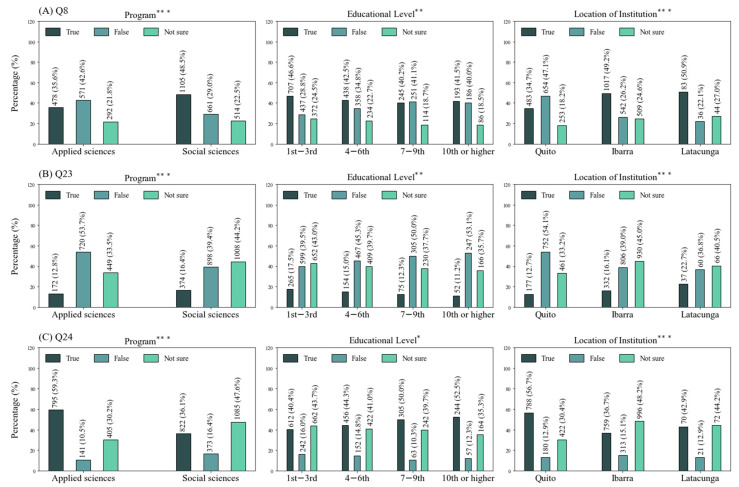
Percentages of questions answered correctly by less than half of the population. (**A**) Question 8; (**B**) Question 23; (**C**) Question 24. * Chi-square values significant at *p* < 0.05; ** at *p* < 0.01; *** at *p* < 0.001.

In general, most participants were knowledgeable about the main clinical symptoms of COVID-19. However, students were not so accurate with regards to symptoms less frequently associated with the disease than with the common cold. Similarly, interviewees were indeed aware of the differences in specificity between detection tests. The majority of them recognized that there is no effective cure for the disease and that chlorine dioxide cannot be considered safe. However, only 34.02% agreed that the use of natural alternatives, such as honey, garlic, ginger or eucalyptus, cannot be deemed useful nor effective for treating the disease (Q8) (Table 4). A significant relationship was determined between this misconception and all demographic variables, except sex. Individuals from social sciences had a greater tendency to agree with this misconception, as did younger students and those affiliated with universities in Ibarra and Latacunga. (Figure 1A).

Overall, participants responded correctly to questions dealing with routes of transmission, albeit with some confusion regarding the airborne transmission of the virus (Q13) (Table 4). No significant relationships were found between this affirmation and any demographic variable.

The vast majority of students were accurate with regards to preventive measures (Table 4). On the other hand, participants proved to be less knowledgeable when asked about SARS-CoV-2. For instance, only 44.68% were aware that the virus could not be observed using a common microscope (Q23). Similarly, only 44% knew that the agent responsible for COVID-19 is an RNA virus (Q24) (Table 4). Significant relations were found between these misconceptions and study program, educational level and location of institution. Students from social sciences were more prone to confusion with regards to these matters, as were younger students and those enrolled in universities located in Ibarra and Latacunga (Figure 1B,C).

**Figure 2:**
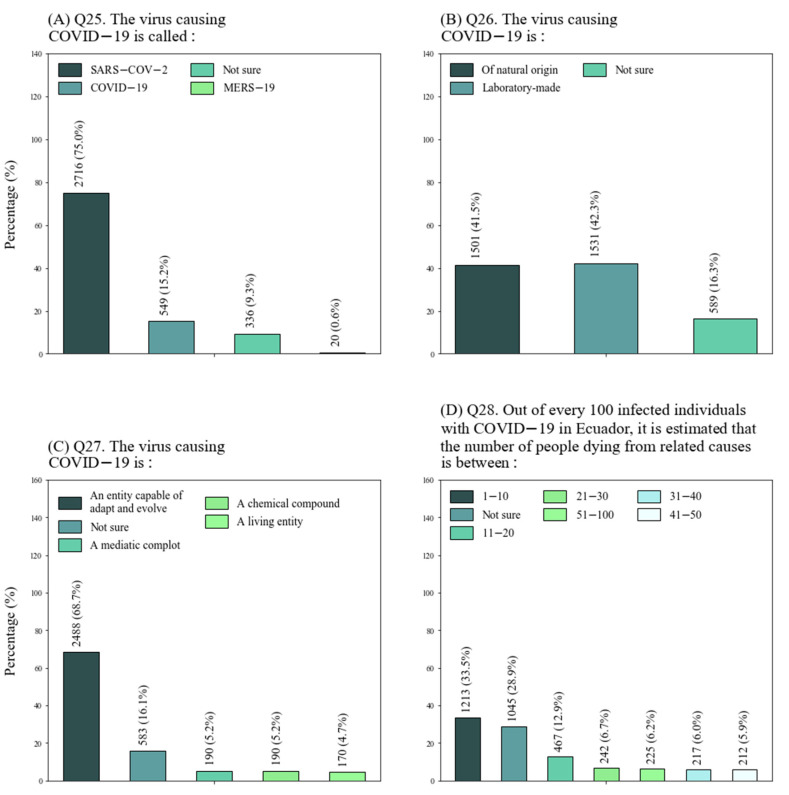
Percentages for multiple-option questions. (**A**) Question 25; (**B**) Question 26; (**C**) Question 27; (**D**) Question 28.

**Figure 3:**
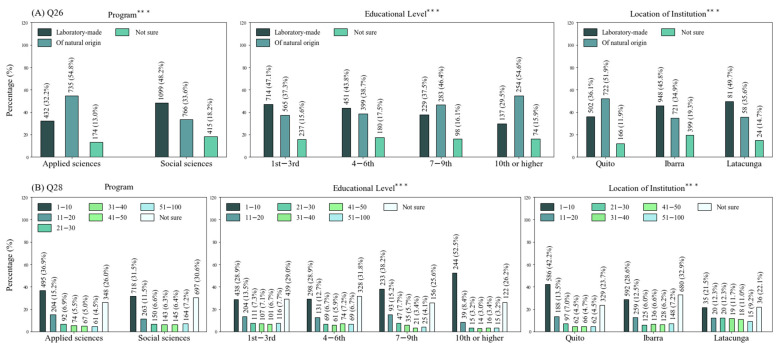
Percentages of multiple-option questions answered correctly by less than half of the population. (**A**) Question 26; (**B**) Question 28. *** Chi-square values significant at *p* < 0.001.

In general, participants identified the name of the virus (Q25) correctly and were more likely to define it as an entity capable of adapting and evolving (Q27), although they had the tendency to believe that the virus was created in a laboratory (Q26) (Figure 2A–C). Significant relations were found between this misconception and all demographics, except for sex. This notion was common among students in social sciences, younger students and individuals affiliated with institutions in Ibarra and Latacunga (Figure 3A). Overall, interviewees were unclear about the fatality rate in the country (Q28), as only 33.5% selected the correct answer (Figure 2D). Again, significant relationships were determined between this question, educational level and location of institution. Younger students, as well as those associated with universities in Ibarra and Latacunga, were more likely to select the “not sure” option (Figure 3B). In brief, these results showed that younger students, generally from social sciences, located in Ibarra and Latacunga, appeared to struggle more with concepts related to the virus. On the other hand, older students from applied sciences and located in Quito were more proficient at answering the questionnaire.

## 4. Discussion

In this research, we determined a correct overall rate of 75.57%, with students from applied sciences scoring higher than those from social sciences. Likewise, students enrolled in later semesters obtained higher scores than younger ones. These differences could not be considered large. In general, participants were knowledgeable about the detection and prevention of COVID-19, albeit with uncertainty with regards to symptoms, treatments and transmission. Moreover, students seemed to lack sufficient criteria about the important characteristic of the virus.

Previous studies have evaluated knowledge of COVID-19 among Ecuadorians [20,21]. However, to the best of our knowledge, the present study represents the first comprehensive investigation about COVID-19 in Ecuadorian undergraduate students, with participants coming from different fields of study in various universities. The study involved only students affiliated with institutions of the northern central region of the country, located in the cities of Quito, Ibarra and Latacunga. The fact that national educational institutions have proposed reopening plans [26], combined with the high transmissibility of the disease, places this subpopulation at a higher risk of contracting and spreading the virus. Moreover, some of the participants were medical students (14%) who are commonly referred to for healthcare advice, especially during the current COVID-19 pandemic. Therefore, having a reference study reporting the level of knowledge among undergraduates seems fundamental. In addition, identifying if popular misconceptions are indeed prevalent among students will prove beneficial for future educational interventions and health programs, which could be designed to address deficient knowledge. In the current investigation, students were given the correct responses after answering the questionnaire with the aim of clarifying or rectifying potential misconceptions. After the session, individuals were given reliable information that could be disseminated within their communities. Indeed, most participants provided positive feedback, mentioning that they felt more knowledgeable and confident after taking part in the survey (Table S3). The aim of this approach was to improve COVID-19 literacy among students, as appropriate knowledge is required to promote healthier attitudes. Poor attitudes have been associated with practices that increase infection and inefficient diagnoses, which ultimately lead to the dissemination of infectious diseases [34,35].

Previous studies considered knowledge of COVID-19 to be sufficient when the overall correct rate was higher than 90% and moderate when it was around 80% [22,23,24]. In the present research, the average knowledge score of participants was 21.16 ± 3 out of 28, with a correct rate of 75.57%. Earlier works, using similar surveys, have reported higher rates among Ecuadorian (80.4%), Malaysian (80.5%) and Chinese (90%) citizens [20,23,24]. Similar results have been observed among Colombians (76.8%) and Ecuadorian medical students (≥70.0%) [27,36]. Therefore, the level of knowledge reported here cannot be considered sufficient, which demonstrates that there is plenty of work to be done if COVID-19 literacy is expected to increase in the country.

The statistical analysis revealed that students from programs related to social sciences obtained lower scores than those from careers in applied sciences, whose correct rate was 79.3% (22.2 ± 3 out of 28). A recent investigation reported COVID-19 knowledge among Ecuadorian medical students; the authors categorized knowledge scores of ≥16 out of 23 possible points as “high knowledge” [27]. Thus, they concluded that knowledge among these students was high. Various investigations have deemed knowledge sufficient when the answer rate was 90% and moderate when it was 80% [22,23,24]. Consequently, and according to these standards, knowledge of COVID-19 among students of careers in applied sciences can be considered, at best, moderate. Noticeably, students following programs in social sciences obtained lower scores and were more prone to agree with popular misconceptions. This suggests that they struggle more than their peers in discerning information about the virus and the disease. Arguably, all undergraduates need reinforcement of issues regarding COVID-19 and, especially, SARS-CoV-2 itself. Similarly, significant differences were found between educational levels. Younger students were not as proficient as their more experienced counterparts; the latter obtained a correct answer rate of 78.6% (22 ± 3 out of 28). Knowledge among older students could be considered moderate. Certainly, they were more educated about the disease than younger students; this has been shown previously as older students proved to be the most knowledgeable among different levels [37,38]. Undoubtedly, students of all levels should be tutored about the disease and virus to give them the opportunity to be more conscious about prevention practices and less prone to adhere to misinformation. Students from universities located in Quito scored higher than their peers from Ibarra and Latacunga. This information appears crucial for designing local actions regarding public health campaigns in areas where knowledge may be inferior.

The present outcomes revealed that students were well-informed about the detection and prevention of COVID-19, although they were less certain with regards to symptoms, treatments and transmission. In general, the sampled population lacked sufficient knowledge about the SARS-CoV-2 virus. Previous studies have also reported that knowledge about symptoms (such as stuffy or runny nose and airborne transmission of the virus) is not necessarily common among the public [20,21,23]. It is important to mention that Q13 might appear problematic to some interviewees as tiny suspended particles can remain airborne for hours, thus constituting an important route of transmission [39]. The low percentage of correct answers associated with this query might be related to this confusion. Arguably, identifying such common patterns in COVID-19 misunderstandings appears crucial for developing future informative campaigns. This study has also highlighted some aspects that should be addressed immediately. First, it is evident that undergraduates are being influenced by misinformation disseminated mainly on social media, as the majority of them thought that natural alternatives could be used to treat the disease. In Ecuador, misinformation has been widely spread via social media concerning subjects such as the origin of the virus, vaccine development and the use of unproven treatments; the latter includes the use of eucalyptus, ginger and seawater [18,19]. In particular, adherence to these alternative treatments was more popular in students affiliated with careers related to social sciences, as well as in those coursing the first semesters. A similar result was observed in a previous study involving Ecuadorians tested for the virus, in which more than 40% of participants gave credit to natural alternative treatments [20]. Since the COVID-19 outbreak, various unproven and uncertified treatments have been reported as effective to battle the effects of infection, albeit lacking reliable information and ignoring potential side effects [40,41]. Furthermore, the consequences left by the disease have worsened attitudes among the public, which has increased the popularity of pseudoscience and alternative medicine [42].

Second, knowledge about the biology of the virus could be considered deficient, as less than half of the population answered correctly the questions regarding the virus RNA composition, its origins and the ability to observe it using a common microscope. This was particularly evident among younger students and those affiliated with programs in social sciences. It is important to mention that most of these students selected the “not sure” alternative, which reinforces the notion that students may not be properly trained on the subject. An analogous situation has been hitherto described among Ecuadorians [20]. In this case, half of the sampled population provided correct responses, while incorrect answers were, in their majority, related to the “not sure” option. It has been determined that undergraduate students associated with careers unrelated to life sciences in the city of Quito were not necessarily aware of basic genetic concepts such as the relation of genes to proteins and the number of chromosomes inherited from each progenitor [25]. Arguably, future educational programs should teach students basic genetic concepts to improve their capacity to discern crucial subjects, including transmission, infection and vaccination. Moreover, the notion that the virus has been created in a laboratory has proved popular among Ecuadorians [20]. Interestingly, this concept proved to be common among all undergraduates, including those affiliated with programs in applied sciences, suggesting that the public is inclined towards the theory of a man-made virus, excluding more reasonable interpretations considering processes of natural selection [43,44]. Undoubtedly, further educational campaigns should bestow individuals with information on the origin of the virus to help people improve their knowledge regarding potential future zoonotic events.

Finally, only 33.5% of the sampled population accurately estimated the national fatality rate per 100 individuals; official sources claim this to be around 5.4% [12]. A previous report demonstrated that this notion was not common among individuals tested for the virus in the cities of Quito and Ibarra, Ecuador [20]. The next most common answer was “not sure”. Thus, it appears that the public is not familiar with the national fatality rate. Not knowing this information might cause people to over- or underestimate the number of deaths per hundred individuals, which could undermine the public’s ability to understand and practice appropriate behaviors to reduce the dissemination of the virus. It is recommended to include this information in further health campaigns.

In general, research assessing the knowledge of COVID-19 among university students has revealed that information regarding symptoms, infection and prevention strategies are well understood. However, such studies did not include questions about the origin of the virus, its characteristics and the efficacy of unproven treatments [27,38,45,46,47]. The current report shows that these subjects are not entirely understood by undergraduate students and so must be included in further investigations. The insufficient knowledge regarding the virus’ main characteristics, along with the constant bombardment of misinformation, could be held responsible for the popularity of misconceptions; these may obstruct the development of a proper interpretation of the disease among the public, which is fundamental in preventative strategies and vaccination campaigns. Based on the present outcomes, we suggest that national health authorities, together with universities, implement educational programs providing basic genetic information not only regarding viral infections but also other diseases. These programs should be implemented as required credit courses, especially for those students following careers in social sciences. Certainly, enhancing the literacy of COVID-19 will contribute to the promotion of protective health measures aimed at containing the spread of this contagious disease.

This study has some limitations. First, the present research was limited due to its cross-sectional design, which does not allow the establishment of causal inferences. Moreover, the fact that the study is based on voluntary responses may overestimate the actual knowledge about COVID-19. Further research must be carried out using probability sampling in order to generate representative results. Second, the survey was carried out in Quito, Ibarra and Latacunga; thus, the obtained results are not generalizable to other regions of the country, especially as national standards of education are not standardized across the country. As previously stated, this research did not intend to generate data that could be extrapolated to other groups. Instead, it sought to develop an initial understanding of COVID-19 knowledge among undergraduates that could be used for designing a methodology to assess knowledge at a national level. Third, as we wanted to focus on assessing knowledge of the pandemic in a national context, we used a questionnaire that only evaluates knowledge. Thus, additional efforts must consider perspectives and attitudes in order to determine the actual extent of knowledge, attitudes and perspectives (KAPs) in the general student population. Finally, this study did not contain a section regarding sources of information about COVID-19; such a section must be included in further investigations to contribute to generating more educative health campaigns.

## 5. Conclusions

This investigation provides a comprehensive analysis of COVID-19 knowledge among undergraduates in north-central Ecuador. The overall knowledge score was 75.5%, which cannot be considered sufficient. Participants showed extensive knowledge of most questions regarding detection, transmission and prevention, although they were not fully confident with regards to the virus’ origins, composition and unproven treatments. Unexpectedly, 42% of the students believed that the virus was produced in a laboratory, which was independent of their level of education. These misconceptions may limit the impact of prevention campaigns. Thus, we recommend that educational programs must address such deficient knowledge. Certainly, carrying out these surveys, in which correct answers are revealed at the end of the questionnaire, will certainly improve the literacy among interviewees, which appears fundamental for the public to adhere to prevention practices. Arguably, these results are helpful to evaluate the current situation and apply educational health programs aimed at counteracting the effects of COVID-19.

## Figures and Tables

**Table 1:** Demographics of students and scores.

Variables	Number of Participants	%	Mean Score (Maximum 28)	t	F	*p*	Cohen’s *d*	95% CI *		*η*^2^—(Partial) Eta Squared	95% CI **	
Sex				3.2		<0.01	0.11	0.04	0.18			
Female	2188	60.43	21.00 ± 4									
Male	1433	39.57	21.40 ± 8									
Program				14.7		<0.001	0.50	0.43	0.57			
Careers in applied sciences	1341	37.03	22.23 ± 3									
Careers in social sciences	2280	62.96	20.53 ± 4									
Educational level					62.7	<0.001				0.05	0.04	0.06
10th or higher semester	465	12.84	22.51 ± 3									
7–9th semester	612	16.85	22.04 ± 3									
4–6th semester	1030	28.44	21.18 ± 3									
1st–3rd semester	1516	41.87	20.38 ± 3									
Location of institution					189.46	<0.001				0.09	0.07	0.10
Quito	1390	38.39	22.49 ± 3									
Ibarra	2068	57.11	20.31 ± 3									
Latacunga	163	4.50	20.61 ± 3									

* Confidence intervals for Cohen’s *d*. ** Confidence intervals for *η*^2^.

**Table 2:** Effect size and statistical significance for Tukey *post-hoc* analysis within demographic variables.

Variables	Mean Difference	SE Difference	t	*p*	95% CI *		Cohen’s *d*	95% CI **	
Educational level									
10th or higher semester vs. 7–9th semester	−0.47	0.21	−2.21	0.12	−1.02	0.08	0.14	0.02	0.26
10th or higher semester vs. 4–6th semester	−1.33	0.19	−6.88	<0.001	−1.82	−0.83	0.39	0.28	0.50
10th or higher semester vs. 1st–3rd semester	−2.13	0.18	−11.6	<0.001	−2.60	−1.66	0.61	0.51	−0.72
7–9th semester vs. 4-6th semester	−0.86	0.18	−4.86	<0.001	−1.31	−0.40	0.25	0.15	0.35
7–9th semester vs. 1st–3rd semester	−1.66	0.16	−10.0	<0.001	−2.09	−1.24	0.48	0.38	0.57
4–6th semester vs. 1st–3rd semester	−0.80	0.14	−5.76	<0.001	−1.16	−0.45	0.23	0.15	0.31
Location of institution									
Quito vs. Ibarra	−2.18	0.12	−18.6	<0.001	−2.45	−1.91	0.64	0.57	0.71
Quito vs. Latacunga	−1.89	0.28	−6.73	<0.001	−2.54	−1.23	0.62	−0.40	−0.40
Ibarra vs. Latacunga	−0.30	0.28	−1.08	0.25	−0.94	0.34	0.08	0.08	0.24

* Confidence intervals of differences between means. ** Confidence intervals for Cohen’s d.

**Table 3:** Results of the multiple linear regression on factors related to low scores regarding COVID-19 knowledge.

Variables	Coefficient	Standard Error	t	*p*	95% CI *	
Programs						
Careers in applied sciences vs. careers in social sciences	−1.39	0.12	−11.64	<0.001	−1.63	−1.16
Educational level						
10th or higher semester vs. 7–9th semester	−0.48	0.20	−2.42	0.015	−0.88	−0.09
10th or higher semester vs. 4–6th semester	−0.79	0.18	−4.28	<0.001	−1.15	−0.42
10th or higher semester vs. 1st–3rd semester	−1.75	0.17	−10.06	<0.001	−2.09	−1.41
Location of institution						
Quito vs. Ibarra	−1.62	0.11	−13.51	<0.001	−1.85	−1.38
Quito vs. Latacunga	−2.19	0.28	−7.94	<0.001	−2.73	−1.65

* Confidence intervals for regression coefficients.

**Table 4:** True/False questions used to measure COVID-19 knowledge among undergraduates.

	Correct Answers	%	Incorrect Answers	%	Not Sure Answers	%
**Associated symptoms**						
Q1. Unlike the common cold, stuffy nose, runny nose, and sneezing are less common in persons infected with COVID-19.	1899	52.44	870	24.03	852	23.53
Q2. The main clinical symptoms of COVID-19 are fever, fatigue, dry cough, and body aches.	3413	94.26	84	2.32	124	3.42
Q3. Not all persons with COVID-19 will develop severe cases. Only those who are elderly and have chronic illnesses are more likely to be severe cases.	3517	97.13	50	1.38	54	1.49
**Methods of detection**						
Q4. PCR is the most accurate technique for identifying the virus in a patient.	2621	72.38	367	10.14	633	17.48
Q5. The rapid test is the most accurate technique for identifying the virus in a patient.	2879	79.51	349	9.64	293	8.09
**Treatments for the disease**						
Q6. Currently, there is no effective cure for COVID-19, but early symptomatic and supportive treatment can help most patients recover from infection.	3303	91.22	102	2.82	216	5.97
Q7. Chlorine dioxide is considered safe to treat COVID-19.	2611	72.11	349	9.64	661	18.25
Q8. Some natural alternatives such as honey, garlic, ginger or eucalyptus can be used to treat the disease/fight the virus.	1232	34.02	1583	43.72	806	22.26
**Routes of transmission**						
Q9. Eating or touching wild animals would result in infection by the virus.	2768	76.44	322	8.89	531	14.66
Q10. Persons with COVID-19 cannot infect others if they do not have fever.	3358	92.74	93	2.57	170	4.69
Q11. The virus spreads via respiratory droplets of infected individuals.	3296	91.02	91	2.51	234	6.46
Q12. People can be infected and contagious despite having no clear symptoms of the disease.	3488	96.33	44	1.22	89	2.46
Q13. The virus is airborne transmitted.	1649	45.54	1402	38.71	570	15.74
Q14. Officially, dogs and cats are considered of low risk for transmission.	2281	62.99	850	23.47	490	13.53
**Prevention strategies**						
Q15. To prevent the infection by COVID-19, individuals should avoid going to crowded places and avoid taking public transportation.	3529	97.46	53	1.46	39	1.08
Q16. Isolation and treatment of people infected with the virus are considered effective ways to reduce the spread of the virus.	3460	95.55	85	2.35	76	2.10
Q17. It is necessary for children and young adults to take measures to prevent infection by the virus.	3516	97.10	53	1.46	52	1.43
Q18. People who have contact with someone infected with COVID-19 should be immediately isolated in a proper place, the isolation period being 14 days.	3406	94.06	122	3.37	93	2.57
Q19. The use of gloves provides total security against infection.	2847	78.62	464	12.81	310	8.56
Q20. Ordinary residents can wear face masks to prevent infection by the virus.	3508	96.88	47	1.30	66	1.82
Q21. It is correct to return to the workplace without being tested for coronavirus.	3231	89.23	184	5.08	206	5.69
Q22. People who work in public service should always wear protective screens to prevent infection.	3434	94.84	61	1.68	126	3.48
**Knowledge of SARS-CoV-2**						
Q23. Coronavirus is observable with a common microscope.	1618	44.68	546	15.08	1457	40.24
Q24. The agent responsible for COVID-19 is an RNA virus.	1617	44.66	514	14.19	1490	41.15

## Data Availability

The data presented in this study are available on request from the corresponding author.

## Supplementary Materials

The following are available online at https://www.mdpi.com/article/10.3390/ijerph18168706/s1, Table S1: Questionnaire, Table S2: CHERRIES, Table S3: Raw data.
